# Comparison between head rotation and standard techniques for i-gel™ insertion: a randomized controlled trial

**DOI:** 10.1186/s12871-024-02621-7

**Published:** 2024-07-10

**Authors:** Seohee Lee, Karam Nam, Sang Joon Park, Jae-Woo Ju, Youn Joung Cho, Yunseok Jeon

**Affiliations:** 1 Department of Anesthesiology and Pain Medicine, Seoul National University Hospital, Seoul National University College of Medicine, 101 Daehak-ro, Jongno-gu, Seoul, 03080 Republic of Korea; 2 Department of Anesthesiology and Pain Medicine, Ajou University Medical Center, Ajou University of College of Medicine, Suwon, Gyeonggi Province Korea

**Keywords:** Airway management, Supraglottic airway, Head rotation, I-gel™ insertion, Standard technique

## Abstract

**Background:**

This study evaluated the effect of head rotation on the first-attempt success rate of i-gel insertion, aiming to alleviate the effect of gravity on the tongue and reduce resistance between the device and the tongue.

**Methods:**

Adult surgical patients were randomized to standard and head rotation technique groups. In the head rotation technique group, patients’ heads were maximally rotated to the left before i-gel insertion. The primary endpoint was the first-attempt success rate. Secondary endpoints included the success rate within two attempts (using the allocated technique), time required for successful i-gel placement within two attempts, and success rate at the third attempt (using the opposite technique).

**Results:**

Among 158 patients, the head rotation technique group showed a significantly higher first-attempt success rate (60/80, 75.0%) compared to the standard technique group (45/78, 57.7%; *P* = 0.021). The success rate within two attempts was similar between the groups (95.0% vs. 91.0%, *P* = 0.326). The time required for successful i-gel placement was significantly shorter in the head rotation technique (mean [SD], 13.4 [3.7] s vs. 16.3 [7.8] s; *P* = 0.030). When the head rotation technique failed, the standard technique also failed in all cases (*n* = 4), whereas the head rotation technique succeeded in five out of the seven patients where the standard technique failed.

**Conclusions:**

The head rotation technique significantly improved the first-attempt success rate and reduced the time required for successful i-gel insertion. It was effective when the standard technique failed. The head rotation technique may be an effective primary or alternative method for i-gel insertion.

**Clinical trial registration:**

ClinicalTrials.gov (NCT05201339).

## Background

Supraglottic airway (SGA) devices are extensively used in daily anesthesia practice and emergency airway management owing to their ease of insertion [1, 2]. Among these devices, the i-gel™, a second-generation SGA device, stands out because of its unique features, including a softer composition and no need for air inflation [3]. However, a modest success rate of i-gel insertion on the first attempt, ranging from 54 to 78% has been reported [1, 4]. Advancement of the device may be impeded before reaching the oropharynx by posterior displacement and folding of the tongue when using the standard insertion technique [5, 6]. This impingement could potentially contribute to the failure of i-gel insertion.

A rotation technique involving a 90-degree counter-clockwise rotation of the i-gel™ during insertion was devised [3] to address posterior tongue displacement and folding [5]. Kim and al. demonstrated that the first-attempt success rate was substantially higher with the rotational technique than that with the standard technique (97% vs. 86%) [3]. Furthermore, a meta-analysis corroborated these findings, showing a significantly higher first-attempt success rate for the insertion of various SGA devices using the rotation technique [7]. However, the rotation technique may pose challenges for novices as it entails additional manipulation of the i-gel beyond the standard technique. To offer various options for insertion techniques, simpler methods need to be developed. An increase in the cross-sectional area of the upper airway was observed following head rotation, specifically within the retroglossal airway region between the hard palate and epiglottis [8, 9]. Consequently, employing a head rotation maneuver may ease the progression of the i-gel into the oropharynx by mitigating gravitational impact on the tongue.

Therefore, we hypothesized that rotating the head facilitates i-gel insertion. In this randomized study, we assessed whether the head rotation maneuver improves the first-attempt success rate of i-gel insertion compared to the standard insertion technique.

## Methods

After obtaining approval from the Institutional Review Board of Seoul National University Hospital (approval no. 2111-156-1276), the study protocol was registered with ClinicalTrials.gov (NCT05201339). This single-center, single-blinded, randomized study was performed between December 2021 and May 2023 in compliance with the Declaration of Helsinki and Good Clinical Practice guidelines.

All adult patients (≥ 19 years old) scheduled to undergo surgery after i-gel insertion were included in this study. The exclusion criteria included: refusal to participate, day surgery, preexisting neurological or cognitive deficits, use of antipsychotic medications, a body mass index > 35 kg/m^2^, mouth opening < 2.5 cm, acute sore throat, risk of aspiration (such as, pregnancy, gastroesophageal reflux disease, and hiatus hernia), history of difficult airway, and limited cervical mobilization (such as, atlantoaxial subluxation and history of cervical spine surgery or head and neck surgery). After obtaining written informed consent, patients were randomly classified in the standard and head rotation technique groups using a computer-generated random number table with a block size of two or four.

Upon patient arrival in the operating room, an attending anesthesiologist blinded to the group allocation assessed the Mallampati score, interincisal distance, and thyromental distance. After denitrogenation with 100% oxygen, general anesthesia was induced and 20–30 mg rocuronium was administered intravenously to facilitate i-gel insertion. After 90 s of manual ventilation, a board-certified anesthesiologist with > 7 years of experience in SGA device insertion (J.-W.J) inserted a gel-lubricated i-gel (Intersurgical Ltd., Wokingham, Berkshire, UK). Size 3 i-gels were used for patients weighing 30–60 kg, whereas size 4 were used for patients weighing 60–90 kg. The head rotation technique was devised by the anesthesiologist as a breakthrough maneuver based on personal clinical experience to address difficulties encountered with the standard technique.

In the standard technique group, i-gel insertion was performed by the operator according to the manufacturer’s instructions. The i-gel was introduced into the oral cavity along the hard palate in the sniffing position with the head unrotated. Pushing posteriorly, the i-gel was gently advanced along the hard palate, soft palate, and oropharynx to the hypopharynx until resistance was experienced. In the head rotation group, the heads of patients were rotated to the left to the maximum before inserting i-gel from the right side of the tongue, which was allowed to descend downward due to gravity. Further, it was gently advanced until the i-gel reached the soft palate and oropharynx, indicating it had reached the level of the tongue base. Thereafter, the head was returned to a neutral position. Subsequently, the i-gel was advanced further into the hypopharynx until resistance was encountered.

If advancement of the i-gel was impeded in both groups before reaching the hypopharynx, the i-gel was withdrawn and reinserted. If insertion failed despite this second attempt, it was recorded as a ‘failure using the allocated technique’, and the operator made a third attempt using the opposite insertion technique. A research assistant assessed successful i-gel placement based on a square-wave capnogram and the absence of an audible leak at a peak airway pressure ≥ 10 cmH_2_O [4]. If deemed improperly placed despite various manipulations such as slight advancement or withdrawal of the i-gel, neck extension, or flexion, the insertion attempt was considered a failure. Each attempt was limited to a maximum of 60 s. The research assistant recorded the number of attempts and the time required to achieve successful placement.

If the third attempt was unsuccessful, the i-gel was inserted with the aid of an assistant performing jaw thrust and deep rotation, following the manufacturer’s recommendations (i.e. a fourth attempt). Tracheal intubation was performed if i-gel insertion was unsuccessful after four consecutive attempts.

The primary outcome of this study was the first-attempt success rate of i-gel insertion. The secondary outcomes were the success rate within two attempts (using the allocated insertion technique), time required for successful i-gel placement within two attempts (from the moment the i-gel was first entered into the oral cavity until a square-wave capnogram was observed and the absence of an audible leak at a peak airway pressure ≥ 10 cmH_2_O was confirmed), success rate at the third attempt (using the opposite technique), conversion to tracheal intubation, and procedure-related complications (sore throat and hoarseness) assessed during the post-anesthesia care unit stay. These outcomes were assessed and recorded by an attending anesthesiologist in the operating room and a research assistant nurse in the post-anesthesia care unit, who were blinded to the group allocation and purpose of the study. The sample size was calculated on the basis of a previous study that reported a 78% first-attempt success rate for i-gel insertion using the standard technique [1]. To detect a 20% increase in success rate with a significance level (α) of 0.05 and power (1 – β) of 0.8 while considering a dropout rate of 10%, each group required 86 participants.

All data are presented as the mean (standard deviation), median (interquartile range), or number (proportion), as appropriate. The independent t-test or Mann–Whitney U-test was used to compare continuous variables, with a prior check for normal distribution using the Kolmogorov–Smirnov test. Categorical variables were compared using the chi-squared test or Fisher’s exact test, as appropriate. The P-values for the secondary outcomes were false discovery rate-adjusted to address multiple testing. SPSS version 23 (IBM, Armonk, NY, USA) was used for all the statistical analyses. Statistical significance was set at *P* < 0.05.

## Results

**Fig. 1:**
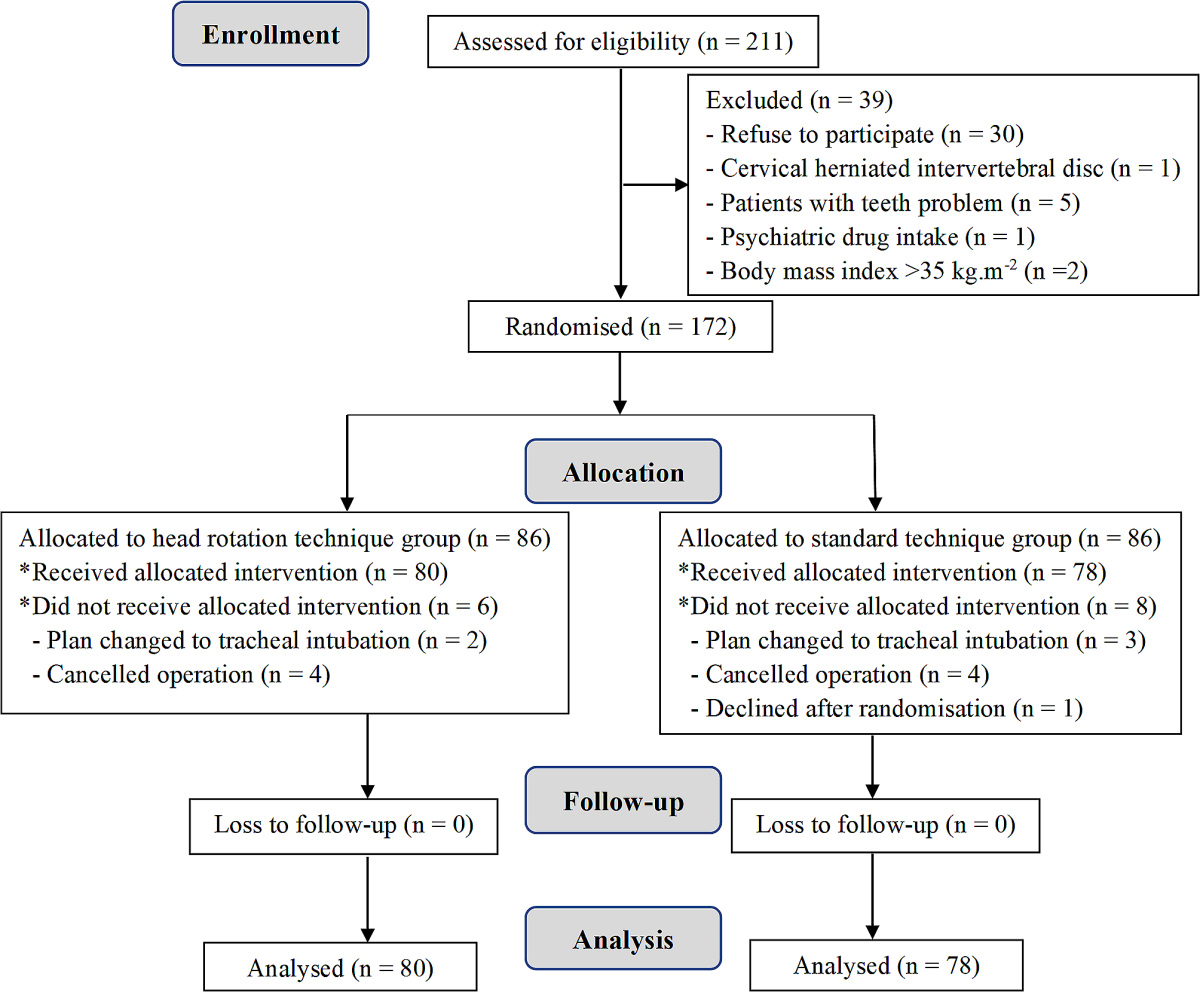
CONSORT flowchart of the study

In total, 211 patients were screened for eligibility, and 39 were excluded. Subsequently, 172 patients were randomly classified into the study groups. Six patients in the head rotation technique group and eight in the standard technique group dropped out. Finally, 158 patients were included in the analysis: 80 in the head rotation technique group and 78 in the standard technique group (Fig. 1). No notable differences in the demographics or airway profiles between the two groups were observed (Table 1).

**Table 1:** Patient demographics and airway profiles

	Head rotation technique (*n* = 80)	Standard technique (*n* = 78)	ASD^*^
Age (years)	58 (48–66)	59 (50–68)	0.145
Female	65 (81.3%)	60 (76.9%)	0.106
Height (cm)	159.5 (7.1)	162.3 (13.5)	0.259
Weight (kg)	62.2 (10.9)	62.6 (9.4)	0.036
Body mass index (kg/m^2^)	24.4 (3.4)	24.1 (3.2)	0.079
Inter-incisor distance (cm)	4.9 (4.3–5.2)	5.0 (4.5–5.2)	0.148
Thyromental distance (cm)	7.5 (7.2–8.0)	7.3 (7.1–7.7)	0.076
Mallampati score			0.046
I	56 (70.0%)	57 (73.1%)	
II	15 (18.8%)	19 (24.4%)	
III	9 (11.3%)	2 (2.6%)	
IV	0 (0%)	0 (0%)	

Data are presented as mean (standard deviation), median (IQR), or number (proportion). ASD, absolute standardized difference

^*^Standardized difference was calculated using Cohen’s *d*, where the difference in means or proportions was divided by the pooled standard deviation

The results of i-gel insertion are presented in Table 2. The first-attempt success rate was significantly higher in the head rotation technique group compared with the standard technique group (75.0% vs. 57.7%; odds ratio, 2.20; 95% confidence interval, 1.12–4.33; *P* = 0.021). The overall success rate within two attempts (using the allocated technique) was comparable between the study groups (95.0% vs. 91.0%, *P* = 0.326). The mean (standard deviation) time required for successful i-gel placement (within two attempts using the allocated technique) was significantly shorter in the head rotation technique group (13.4 [3.7] s) than that in the standard technique group (16.3 [7.8] s; *P* = 0.030). I-gel insertion using the standard technique was also unsuccessful in all patients (*n* = 4) in whom the head rotation technique failed. However, among seven patients in whom the standard technique failed, the i-gel was successfully placed using the head rotation technique in five patients. No patient in either group required conversion to tracheal intubation. The incidences of sore throat and hoarseness were not statistically different between the two groups.

**Table 2:** Procedural results according to the study groups

	Head rotation technique (*n* = 80)	Standard technique (*n* = 78)	*P*
The primary outcome			
First-attempt success rate	60 (75.0%)	45 (57.7%)	0.021
The secondary outcomes^*^			
Overall success rate (within two attempts)^†^	76 (95.0%)	71 (91.0%)	0.326
Time to successful placement (s)^‡^	13.4 (3.7)	16.3 (7.8)	0.030
Third-attempt success rate using the opposite technique	0/4 (0%)	5/7 (71.4%)	0.102
Conversion to tracheal intubation	0 (0%)	0 (0%)	NA
Sore throat	14 (17.5%)	9 (11.5%)	0.326
Hoarseness	1 (1.3%)	7 (9.0%)	0.083

Data are presented as number (%) or mean (standard deviation). NA, not applicable

^*^P values for the secondary outcomes were false discovery rate-adjusted

^†^Using the allocated technique

^‡^Compared in patients in whom the insertion was successful within two attempts using the allocated technique

## Discussion

In this randomized trial, the head rotation technique significantly improved the first-attempt success rate of i-gel insertion compared with that of the standard technique, and a significantly shorter time was required for successful i-gel placement using the head rotation technique. Moreover, in patients in whom i-gel insertion using the standard technique failed, the head rotation technique proved to be a valuable alternative. Conversely, in patients for whom the head rotation technique did not work, the standard technique was also unsuccessful.

The primary cause of airway obstruction in anaesthetized paralyzed patients is the gravitational effect on the anterior upper airway structures in the supine position [9, 10]. The soft palate and tongue may be more susceptible to gravitational forces owing to the lack of rigid bone support. Lateral positional changes reduce upper airway collapse [8, 11]. Similarly, head rotation has been shown to increase the cross-sectional area of the upper airway, thereby improving airway patency in both the awake [8] and sedated supine positions [9]. The head rotation maneuver enhances the effectiveness of mask ventilation, emphasizing the clinical significance of maintaining airway patency by reducing the influence of gravity [12]. This is particularly important as improved airway patency can facilitate easier insertion of airway devices. Based on these considerations, we hypothesized that head rotation may facilitate i-gel insertion by reducing the resistance between the device and the tongue, thereby mitigating gravitational impact. Our study demonstrated a significant improvement in the first-attempt success rate of i-gel insertion using the head rotation technique, supporting this hypothesis.

None of the participants in the head rotation technique group achieved success when switching to the standard technique in the third attempt (0/4, 0.0% success rate). However, in the standard technique group, five of seven participants had successful i-gel insertion in the third attempt using the head rotation technique. This suggests that the head rotation technique can be valuable alternative when the standard technique fails. Although this study did not employ a crossover design, which compares treatment effects within subjects to minimize intersubject variability, [13] the head rotation technique may be considered an option when the standard technique fails. Furthermore, the time to successful mechanical ventilation was significantly shorter in the head rotation group than in the standard technique group. This reduced time may reflect the benefit of the head rotation technique in minimizing manipulation and facilitating more efficient i-gel placement.

While our study supports the head rotation technique as a valuable alternative, further research is required to improve patient outcomes comprehensively. For instance, the head rotation technique may enhance airway management efficiency and safety, particularly in challenging cases such as emergency situations or patients with anatomical variations. Future research should validate these findings across these diverse clinical settings. Additionally, exploring its applicability to other supraglottic airway devices and investigating the underlying mechanisms through imaging studies can further optimize its integration into clinical practice.

This study had some limitations. First, owing to the nature of the study design, the practitioner could not be blinded to the group assignment. Although the practitioner was unaware of the primary endpoint, this may have produced a bias in the results. However, we mitigated this potential bias by blinding the outcome assessors and evaluators to the group allocation and the purpose of the study. Second, all i-gel insertions were performed by a single experienced practitioner. This eliminates the risk of inter-practitioner variability affecting the results; however, limitations in applying the findings to novices or anesthetists who are unfamiliar with the head rotation technique may persist. Third, we did not assess the Cormack–Lehane grade, which may have affected the success rate of i-gel insertion [14]. However, it is likely to have little or no effect due to randomization. Instead, we evaluated airway profiles, such as the Mallampati score, mouth opening (interincisal distance), and thyromental distance, which are recognized as predictors of a difficult airway [15–17]. Nonetheless, the limited inclusion of patients anticipated to have a difficult airway posed a challenge in generalizing the findings of this study to this specific subgroup.

## Conclusions

In conclusion, the head rotation technique demonstrated a significantly higher first-attempt success rate of i-gel insertion and a shorter time to successful placement, with an incidence of complications comparable to that of the standard insertion technique. Therefore, the head rotation technique may be considered as an alternative method for i-gel insertion when the standard technique proves unsuccessful, or even as the primary method.

## Data Availability

No datasets were generated or analysed during the current study.

## Abbreviations

SGA: Supraglottic airway
